# Ecofriendly micellar mediated spectrofluorimetric method for ultrasensitive quantification of the antiparkinsonian drug safinamide in pharmaceutical formulation and spiked human plasma

**DOI:** 10.1038/s41598-024-66462-7

**Published:** 2024-07-16

**Authors:** Engy A. Ibrahim, Hoda M. Marzouk, Maha A. Hegazy, Laila E. Abdel Fattah, Samah S. Saad

**Affiliations:** 1 Pharmaceutical Analytical Chemistry Department, College of Pharmaceutical Sciences and Drug Manufacturing, Misr University for Science and Technology, 6th of October City, Giza, Egypt; 2 Pharmaceutical Analytical Chemistry Department, Faculty of Pharmacy, Cairo University, Kasr Al-Aini Street, Cairo, 11562 Egypt; 3 Pharmaceutical Chemistry Department, Faculty of Pharmacy, Future University in Egypt, Cairo, 11835 Egypt

**Keywords:** Blueness estimation, Greenness assessment, Micellar-based spectrofluorimetric method, Parkinsonism add-on therapy, Safinamide mesylate, Synthetic precursor impurity, Whiteness evaluation, Chemistry, Materials science

## Abstract

A novel, highly sensitive and eco-friendly micellar-mediated spectrofluorimetric method was developed and validated for the determination of the novel antiparkinsonian drug safinamide mesylate in the presence of its related precursor impurity, 4-hydroxybenzaldehyde. The proposed approach relies on increasing the inherent fluorescence emission at 296 nm of safinamide, by forming hydrogen bonds between the mentioned drug and sodium dodecyl sulfate in the micellar system using 0.1 N HCl as a solvent, following excitation at 226 nm. A thorough investigation was conducted into the experimental factors affecting spectrofluorimetric behavior of the studied drug. A linearity plot of safinamide over the concentration range of 10.0–1000.0 ng/mL against the relative fluorescence intensities was established. The proposed method demonstrated excellent sensitivity down to the nano-gram level with detection and quantitation limits of 1.91 and 5.79 ng/mL, respectively. The studied drug was effectively determined in Parkimedine^®^ Tablets. Furthermore, the proposed method allows for ultrasensitive quantification of safinamide in spiked human plasma, with satisfactory percentage recovery (98.97–102.28%). Additionally, the greenness assessment using the advanced green certificate classification approach, the complementary green analytical procedure index (Complex-GAPI), and the analytical GREEness metric approach (AGREE), along with the practicality check using the Blue Applicability Grade Index in addition to the all-inclusive overall whiteness evaluation using the RGB-12 model were carried out. The outcomes demonstrated the effectiveness and whiteness of the proposed technique. Clearly, the suggested approach has the advantages of being simple, requiring no pretreatment steps, and relying solely on direct measuring procedures.

## Introduction

Spectroflourimetry is one of the most popular and emerging analytical techniques for the quantification of compounds in different analytical matrices due to its exceptional high sensitivity. Moreover, it is characterized by its superior rapidity and simplicity, making it widely used for quantitative routine analysis in quality control (QC) laboratories^1^. Due to its capacity to achieve lower detection limits, fluorescence spectrometry is frequently used to quantify trace pharmaceutical compounds in both dosage forms and biological samples^2^. The use of different types of surfactants to boost the fluorescence of various drugs has found several applications in the field of analysis^3–5^. Surfactants are amphiphilic organic molecules with a polar head group, which may be either ionic or neutral and a long non-polar tail. Upon dissolution, these molecules undergo spontaneous aggregation. When reaching a specific concentration known as the critical micellar concentration (CMC), it will form organized molecular assemblies called micelles. The capacity of these organized media to create a suitable microenvironment capable of altering the catalytic and luminescent characteristics of analytes is widely recognized. In addition, surfactants exhibit a lower environmental and human health impact than conventional organic solvents^6–9^. Using sodium dodecyl sulfate (SDS) as a micellar system was particularly effective in incorporating the hydrophobic compounds into the hydrophobic portion of the surfactant micelle in the aqueous solution which greatly enhanced the sensitivity of the method^10–13^.

The progressive loss of dopamine neurons characterizes Parkinson's disease (PD). As a result, dopaminergic drug substitution therapy is the primary therapeutic approach. The gold standard treatment for Parkinson's disease motor symptoms is levodopa^14^. Parkinson's disease requires an increase in levodopa dosage, which can result in motor complications like wearing-off and dyskinesias with long-term and high-dose treatments. So, therapeutic drugs like dopamine agonists and monoamine oxidase B (MAO-B) inhibitors, such as safinamide, have been approved by both the European Medicine Agency (EMA) and the US-FDA to increase levodopa dosage and enhance motor control in PD^15^. There is ongoing research exploring a possible connection between contracting SARS-CoV-2 and developing Parkinsonism. While the exact mechanism by which COVID-19 could contribute to neurodegeneration is not completely understood, some evidence suggests that there may be a causal link between the two^16,17^.

**Figure 1:**
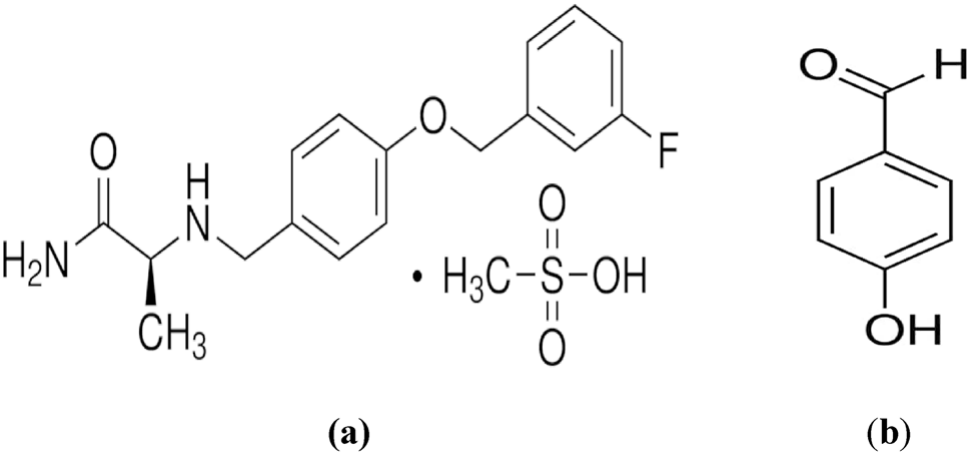
Chemical structures of (**a**) safinamide mesylate (SAF), **(b)** 4-hydroxybenzaldehyde (4-HBD).

Safinamide (SAF) is a reversible, selective inhibitor of both MAO-B and sodium channels that has shown efficacy as an adjunct to levodopa in mid-to-late-stage PD. SAF's dual dopaminergic and glutamatergic mechanism of action reduces OFF time, increases ON time, and improves non-motor symptoms like pain, sleep disorders, and mood^18^. The chemical name for SAF is (*S*)-2-[[4-[(3-fluorophenyl)methoxy]phenyl]methyl]aminopropanamide methane sulfonate^19,20^. The synthetic precursor of SAF is 4-hydroxybenzaldehyde (4-HBD), as shown in Fig. 1^21^.

A thorough review of the literature has identified a number of analytical approaches, such as HPLC^21–26^, HPTLC^27^, UPLC^28^, electrochemical^29,30^, and UV-spectrophotometric methods^31^ that can accurately quantify SAF alone or in combination with other drugs. To our knowledge, there is currently no eco-friendly spectrofluorimetric method for determining SAF in the presence of its related impurity.

Green analytical chemistry has gained significant interest since the 1990s. The immense success and importance of this field lies in the fact that green methods are generally faster, consume fewer reagents and solvents, and reduce or even eliminate waste generation. Therefore, there is an ongoing trend towards substituting traditional methods of analysis with greener options^32–36^. In 2021, White analytical chemistry (WAC) was developed as an extension of the GAC postulates in order to offer a more comprehensive perspective on factors other than sustainability that affect the method's quality and an objective equilibrium among them^37^. The philosophy of WAC consists of twelve concepts, which are categorized into three sections based on the red, green, and blue (RGB) color scheme^37,38^. Mixing the primary colors results in creating a light beam of "white" color. This makes "white" a suitable term for a well-balanced analytical methodology that is fit for a specific application. In summary, the WAC is regarded as an upgraded version of the GAC because of the improved analytical performance (red) and applicability (blue) of the developed method. An Excel spreadsheet is available online, which can be used to assess the extent of compliance of the analytical method with the WAC assumptions in a quantitative way^37,39^.

A new, simple, selective, sensitive, and eco-friendly spectrofluorimetric method was developed in accordance with the principles of green chemistry to estimate SAF in tablet dosage form and human plasma. The method is based on the formation of a hydrogen bond between sodium dodecyl sulfate (SDS) and SAF as a micellar system, which enhances the fluorescence.

## Experimental

### Apparatus

Fluorescence spectroscopy analysis was developed using RF-6000 spectrofluorometer (Shimadzu, Japan). The samples were analyzed using a 5 nm slit width for excitation and emission, and a 1 cm quartz cell was used. The manipulation and collection of data were obtained using LabSolutions software. A Nahita Centrifuge model 2690/5 (Beriain, Spain) was utilized to speed up the phase separation process. For pH adjustments, a pH glass electrode (Jenway, UK) was used.

### Chemicals and reagents

Pure SAF with a purity of 100.70% was given by EVA Pharma for the Pharmaceutical Industry (Al-Giza, Egypt) as a gift, and 4-HBD with a stated purity of 99% was supplied by Acros Organics, Fisher Scientific (Belgium).

Parkimedine^®^ Tablets are claimed to contain 100 mg of SAF per tablet and are produced by EVA Pharma for the Pharmaceutical Industry (B.N. (10) 2112425).

Methanol of HPLC grade was supplied by Merck (Darmstadt, Germany). Ethanol and acetonitrile of HPLC grade were provided by Fisher Scientific (United Kingdom). Hydrochloric acid (HCl) fuming 37%, sodium hydroxide, triton X-100 and tween 80 were provided by PioChem Co. (Giza, Egypt). Different solvents of analytical grade as acetone, butanol, and ethyl acetate were used and supplied by PioChem Co. (Giza, Egypt). Sodium dodecyl sulfate (SDS) was purchased from Advent Chembio Pvt. Ltd. (India). Carboxymethylcellulose (CMC), acetic, phosphoric, and boric acids were obtained from El-Nasr Chemicals Co. (Cairo, Egypt). Throughout the analysis, double-distilled water was used.

Human plasma was kindly provided by the Blood Bank of Souad Kafafi Hospital (6^th^ October, Egypt). It was stored frozen at -20 °C until the analysis was conducted.

### Standard solution

SAF and 4-HBD stock standard solutions (100,000 ng/mL) were prepared separately by dissolving 10.0 mg of the drug and its impurity powder in 40 mL of methanol and adjusting the volume to 100 mL using the same solvent. SAF (10,000 ng/mL) and 4-HBD (1000 ng/mL) working solutions were prepared by further dilution of the stock solutions with methanol.

### General Procedures

#### Calibration curve construction

Aliquot volumes of the working SAF solution were transferred into a series of 10-mL volumetric flasks. Subsequently, 1.5 mL of 10 mM SDS solution were added and filled the flasks up to the marks with 0.1 N HCl solution. After thoroughly mixing the solutions, the final solutions' fluorescence intensity was measured at 296 nm after excitation at 226 nm. A concurrent blank experiment was carried out. The ultimate concentrations ranged from 10.0 to 1000.0 ng/mL.

#### Assay of SAF in binary mixtures with 4-HBD

Synthetic laboratory-prepared mixtures were prepared by mixing different aliquots of SAF and 4-HBD working solutions in different ratios into a series of 10-mL volumetric flasks. The procedures described under "Calibration curve construction" were followed, and the recovery percent of SAF was calculated using the regression equation.

#### Assay of Parkimedine Tablets dosage form

The process began by weighing ten Parkimedine Tablets of and finely powdering them. Then, an amount of SAF equivalent to 100 mg was weighed and transferred to a 100-mL volumetric flask. It was dissolved in 50 mL of methanol and sonicated for 15 min. The volume was then completed to the mark with methanol to produce a SAF solution with a concentration of 1,000,000 ng/mL and filtered through a 0.45 μm pore size filter. Next, 0.5 mL of the SAF solution was taken and transferred to a 50-mL volumetric flask. It was then diluted with methanol to the mark to produce an SAF solution of 10,000 ng/mL. Further dilution was performed as appropriate, followed by the general procedure described in section "Calibration curve construction".

#### Procedure for spiked human plasma

A series of working SAF solutions were prepared from its stock solution using methanol as a solvent in the 1000.0 – 6500.0 ng/mL range. Into 10-mL centrifugation tubes, 450 µL of plasma was transferred and spiked with 50 µL SAF working solutions. Afterward, the mixtures were vortex-mixed for two min; then, 3 mL of methanol was added and centrifuged at 5000 rpm for 30 min till protein precipitation was complete. After that, one mL of the supernatant solution of each mixture was transferred separately into 10-mL volumetric flasks with 1.5 mL of 10 mM SDS, and the volume was completed with 0.1 N HCl to produce the final concentration range of 100.0–650.0 ng/mL.

## Results and discussion

**Figure 2:**
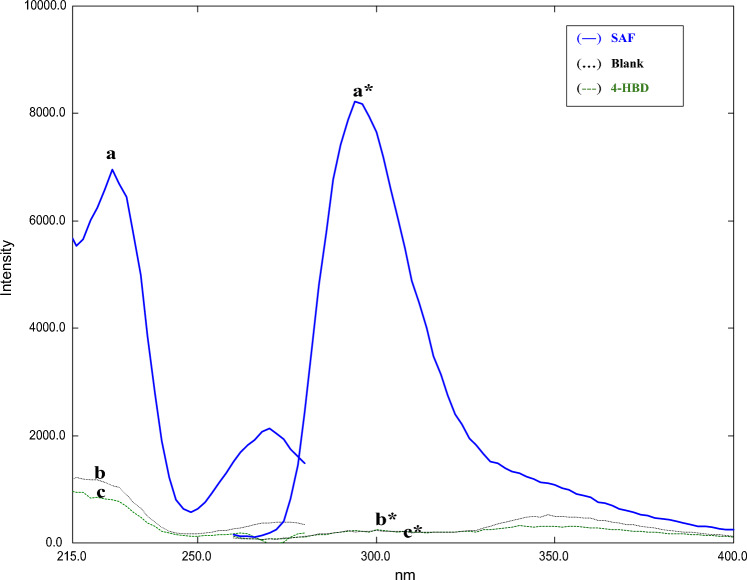
(**a**) Excitation and (a*) emission spectra of SAF (800.0 ng/mL), (**b**) Excitation (**…**) and (b*) emission (**…**) spectra of blank, and (**c**) Excitation (**–**) and (c*) emission (**–**) spectra of 4-HBD (200.0 ng/mL).

SAF was found to exhibit an intense native fluorescence at 296 nm in 0.1 N HCl solution after excitation at 226 nm, as presented in Fig. 2. This work involves employing a micellar media to boost the fluorescence intensity of the studied drug. The goal of this research is to use the emission bands to create a new, highly sensitive, simple, and specific approach for analyzing SAF in its bulk form, pharmaceutical formulations, and spiked human plasma. Various factors that influence the native fluorescence of SAF were examined, such as the solvent used for dilution, the buffer system, and the quantity and concentration of the organized media.

### Effect of diluting solvent

**Figure 3:**
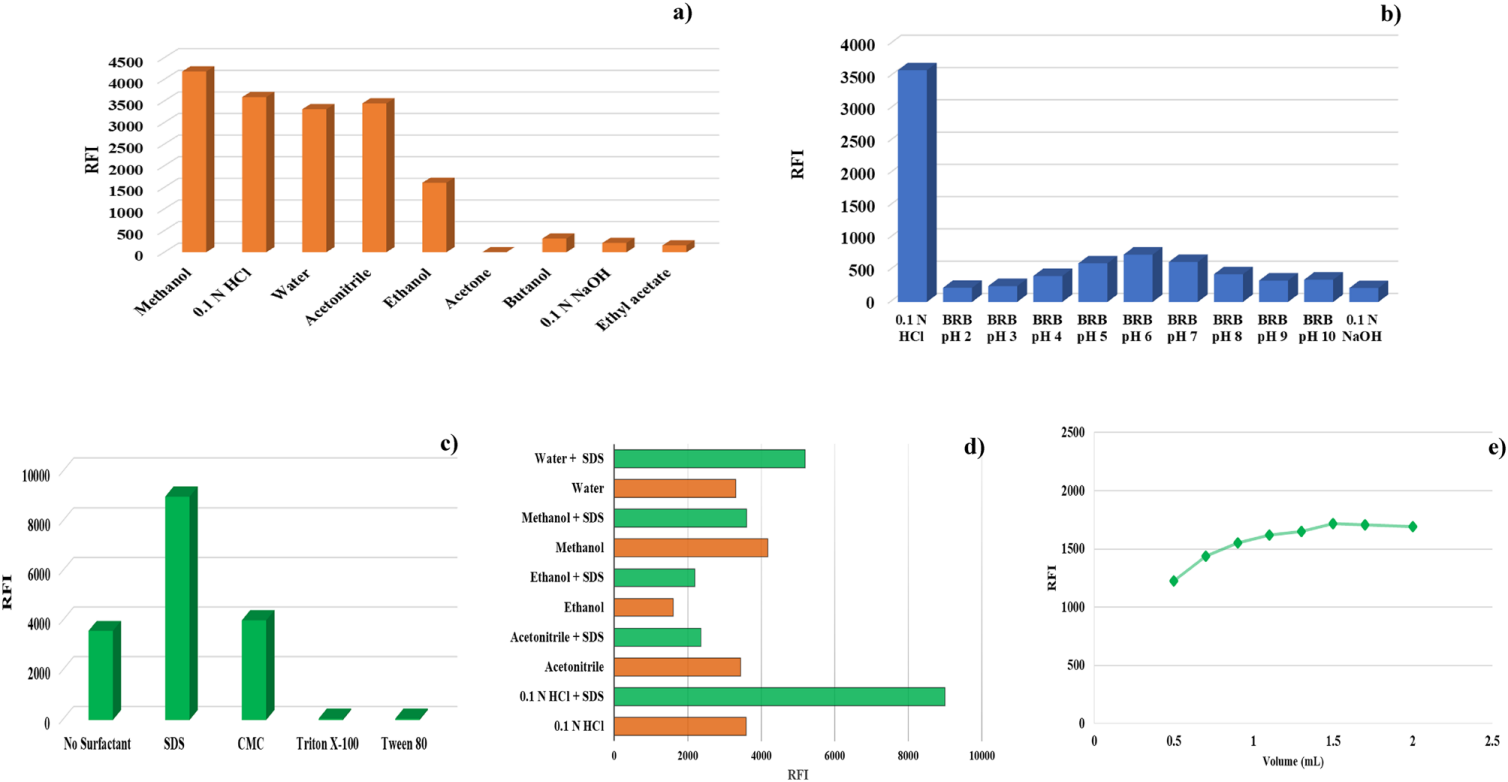
Effect of (**a**) diluting solvents, (**b**) pH, (**c**) organized media, (**d**) different solvents with organized media and (**e**) volume of 10.0 mM SDS on RFI of SAF.

An investigation was conducted into the impact of various diluting solvents on SAF's relative fluorescence intensity (RFI). Acetone, acetonitrile, butanol, ethanol, ethyl acetate, methanol, water, 0.1 N HCl, and 0.1 N NaOH were among the tested solvents. The findings indicated that these solvents could be divided into three groups. The first group of solvents included acetone, butanol, ethyl acetate, and 0.1 N NaOH, all of which quenched SAF's fluorescence. The second group, which minimized the blank fluorescence intensity while increasing the RFI, including 0.1 N HCl, water, and methanol. The final group consisted of ethanol and acetonitrile, demonstrating a non-quantitative RFI and a blunted emission peak. As illustrated in Fig. 3a, methanol showed the maximum SAF RFI followed by 0.1 N HCl among the studied solvents.

### Effect of pH

The fluorescence properties of SAF were studied using 0.04 M Britton-Robinson buffer, 0.1 N NaOH and 0.1 N HCl solutions to cover different pH ranges. Using 0.04 M Britton-Robinson buffer and 0.1 N NaOH showed a noticeable quenching in SAF RFI at the pH range 2–12. SAF fluorescence was found to be more intense in acidic media. As can be seen in Fig. 3b, the best RFI was achieved in 0.1 N HCl.

### Effect of organized media

Different types of surfactants were studied for their ability to enhance the fluorescence intensity of drugs above their critical micelle concentration. Various surfactants were tested individually to determine their effect on the fluorescence intensity of SAF solution. These surfactants included anionic surfactant (SDS), non-ionic surfactant (Tween 80 and Triton X-100), and anionic polysaccharide (CMC sodium). A 2% (w/v) aqueous solution of each surfactant was added to SAF solution in one mL, and the RFI was measured. It was observed that out of all the surfactants, only SDS was able to increase SAF fluorescence intensity to its maximum potential. The ideal concentration of SDS was determined by testing concentrations ranging from 10 to 69 mM (2% w/v). It was discovered that the fluorescence intensity remained constant over the concentration range of 10 mM to 69 mM (2% w/v). The reason behind this enhancement may be attributed to the formation of ion-pair complex between SAF due to the electrostatic attraction between the positively charged amino group of the drug and the negative sulfonyl group of SDS. Micellar binding increased the intensity of SAF fluorescence by reducing free rotation and collisions with the solvent molecules^6^. However, it was observed that Tween-80 and Triton X-100 decrease the RFI, as illustrated in Fig. 3c.

The effect of SDS on SAF RFI was studied with different diluting solvents. SAF RFI increased when SDS was used with 0.1N HCl as a diluting solvent giving the suggested method an extra green advantage, while it decreased with organic solvents, as presented in Fig. 3d. The observed reduction in micelle formation may be attributed to the denaturing effect of organic solvents on the micelle, which alters their properties as they dissolve in water. Additionally, using organic solvents can also lead to a decrease in the size of micelles^40^.

When studying the effect of 10 mM SDS volume, it was found that the addition of 1.5 mL of SDS solution to SAF, followed by completing the volume with 0.1 N HCl as a diluting solvent, resulted in the highest RFI, as shown in Fig. 3e.

## Method validation

The analytical procedure was validated following ICH guidelines^41^. The following parameters were evaluated: linearity, range, LOQ, LOD, accuracy, precision, and robustness.

### Linearity & range

Linear regression was performed to create a standard calibration curve for the studied drug. SAF demonstrated a good linear relationship over the concentration range of 10.0–1000.0 ng/mL, as shown in Fig S1. The regression equation parameters are presented in Table 1.

**Table 1:** Regression parameters and assay validation report of the proposed spectrofluorimetric method for determination of safinamide.

Parameter	SAF
λ_ex_ (nm)	226
λ_em_ (nm)	296
Linearity range (ng/mL)^a^	10.0–1000.0
Slope	12.612
Intercept	288.82
Correlation coefficient (r)	0.9997
Accuracy	
(Mean ± SD)	100.01 ± 1.453
'Precision	
± (%RSD)^b^	1.044
± (%RSD)^c^	1.900
Selectivity^d^	99.37 ± 1.402
LOD (ng/mL)^e^	1.91
LOQ (ng/mL)^e^	5.79
Robustness (%R ± %RSD)	
Wavelength of measurement ± 1 nm	100.98 ± 0.734
SDS volume ± 0.1 mL	± 1.760

^a^Six concentrations of SAF (10.0, 100.0, 400.0, 600.0, 800.0, 1000.0 ng/mL) were used to construct the calibration curve.

^b^Intra-day precision [average of 3 different concentrations of 3 replicate each (n = 9) within the same day].

^c^Inter-day precision [average of 3 different concentrations of 3 replicate each (n = 9) repeated on 3 successive days].

^d^Recovery% (Mean ± SD) of SAF in laboratory-prepared mixtures with its related impurity.

^e^Limit of detection and quantitation are determined via calculations, LOD = (SD of regression residuals/ slope) × 3.3; LOQ = (SD of regression residuals/ slope) × 10.

### Accuracy

The accuracy of the method was assessed by calculating the mean recovery percentage of the pure standard at five different concentrations (50.0, 200.0, 300.0, 500.0, and 700.0 ng/mL). As shown in Table 1, the results obtained were within the acceptable range, demonstrating the high level of accuracy of the recommended method.

### Precision

Intra- and inter-day precisions calculations of the proposed method were assessed by measuring three different SAF concentrations (400.0, 600.0, 800.0 ng/mL), in triplicates, over one day and three consecutive days, respectively. Table 1 displays small values of % RSD, which indicate the precision of the method.

### Detection and quantification limits

Based on the calibration data, the limit of detection (LOD) and limit of quantitation (LOQ) were determined using the standard deviation of residuals (σ) and the slope (s) with the formulas LOD = 3.3 × σ/s and LOQ = 10 × σ/s, respectively, as illustrated in Table 1. Table S1 displays a comparison of the LODs in this work with earlier reported ones. Clearly, the proposed spectrofluorimetric method is characterized by its exceptional sensitivity.

### Selectivity

Analyzing laboratory-prepared mixtures with varying ratios of the drug and its related impurity (4-HBD) was carried out to verify the proposed method's selectivity, Table 1. Results reveal satisfactory recoveries and excellent precision values for different ratios, Table S2.

The method's selectivity was further established by conducting an analysis of a tablet formulation containing SAF. The presence of common tablet excipients and additives did not impact the accuracy of the analysis, as evidenced by the acceptable percentage recoveries achieved in Table 2.

**Table 2:** Estimation of safinamide in Parkimedine tablets by the proposed spectrofluorimetric method and application of standard addition technique.

Drug	Parkimedine^®^ tablets (BN: (10) 2,112,425) (Each tablet labelled to contain 100 mg SAF)		Standard addition technique	
	%Found ± SD^a^	Claimed (ng/mL)	Pure added (ng/mL)	%Recovery of the pure added^b^
SAF	100.71 ± 1.215	200	100.0	100.85
			200.0	99.51
			400.0	99.97
		Mean ± SD		100.11 ± 0.683

^a^Average of five determinations.

^b^Average of three experiments.

### Robustness

The proposed method's robustness was evaluated by repeating the procedure with slight changes to the optimum conditions, such as wavelength of measurement (± 1 nm) and volume of SDS (± 0.1). A single parameter was adjusted slightly, while all other conditions remained unchanged. The slight modifications did not have a significant impact on the fluorescence intensity. In addition, the procedure demonstrated high reliability, as shown by the low % RSD of the responses (< 2%) in Table 1.

### Analysis of pharmaceutical formulation (Parkimedine^®^ Tablets)

The spectrofluorimetric method accurately quantified SAF in Parkimedine^®^ Tablets without separation or interference from excipients. To confirm the validity of the suggested method and to show how well the extraction procedure was performed, the standard addition technique was also used. As shown in Table 2, the results were satisfactory.

### Application in human plasma samples

Once 100 mg of SAF tablet is taken orally, it takes about two h to reach the maximum plasma concentration (C_max_), which was approximately 650 ng/mL^20,23^. The proposed spectrofluorimetric method demonstrated high sensitivity in detecting SAF in spiked human plasma samples with no interference from the endogenous matrix. Various concentrations of SAF were estimated using the regression equation, Y = 3.0905x − 301.09 (r^2^ = 0.9997). The analytical parameters confirmed the accuracy of the method, with an average recovery value of 100.17 ± 1.322, as shown in Table 3.

**Table 3:** Determination of safinamide in spiked human plasma by the proposed method.

Drug	Spiked conc. (ng/mL)	Conc. found (ng/mL)	Recovery (%)^a^
SAF	100	102.28	102.28
	200	198.64	99.32
	300	296.91	98.97
	400	397.80	99.45
	500	506.63	101.33
	650	647.76	99.65
	Mean ± SD		100.17 ± 1.322

^a^Average of three determinations.

### Assessment of the method greenness

#### Green certificate classification approach (GCC)

The Green Certificate-modified Eco-Scale evaluates analytical procedure characteristics such as reagent toxicity, instrumental energy consumption, and toxic compound emissions to the environment, as well as reagent and solvent volumes used and waste generation, which are scored using a penalty-point system^42^. As shown in Table 4, the proposed method is greener than the reported HPLC–UV method^26^.

**Table 4:** Comparative assessment comparison of the proposed spectrofluorimetric method and the reported HPLC-UV method according to GCC, Complex-GAPI, AGREE, BAGI tools and whiteness assessment by RGB 12 model.

Method	GCC tool^a^	Complex-GAPI assessment^b^	AGREE assessment^c^	BAGI assessment^d^	RGB12 algorithm^e^
For proposed spectrofluorimetric method					
For reported HPLC-UV method^26^					

^a^The Green Certificate-modified Eco-Scale evaluates analytical procedure using a penalty-point system^42^.

^b^Complex-GAPI Assessment is an update to the commonly used GAPI metric and evaluated according to Green Analytical Procedure Index parameters description^43^.

^c^AGREE Assessment evaluated by using Analytical GREEnness Metric approach and Software^44^.

^d^Blue Applicability Grade Index (BAGI) is proposed as a new metric tool for evaluating the practicality of an analytical method^45^.

^e^RGB12 algorithm for whiteness evaluation is a unique ideology tool for applying sustainable development concepts in analytical chemistry^37^.

#### Complementary green analytical procedure index (Complex-GAPI)

This is a more recent version of the widely used GAPI metric^43^. To evaluate a system's methodology, the assessment employs a visual representation. Each of the five aspects of the methodology is represented by a pentagram. Sample collection and preparation, reagents and solvents, instrumentation, and method type are all examples of these aspects. The hexagonal field represents the pre-analysis procedures, and the pentagrams represent the overall quality and effectiveness of each aspect. The pre-analysis phase is a crucial component of the entire methodology and significantly impacts the outcomes' accuracy and dependability, so its inclusion is crucial. The assessment's color scheme is noteworthy. It depicts the effects of each stage on the environment using a range of colors. Red denotes actions that could be hazardous to the environment and serve as a warning. The actions in yellow indicate a moderate level of concern and have a moderate impact on the environment. Green highlights environmentally friendly behaviors that have little to no adverse environmental effects. The assessment uses a color scheme and visual representation to give a thorough and understandable assessment of the methodology, particularly emphasizing the methodology's sustainability and impact on the environment. Eight green patches in the suggested approach indicated good greenness (Table 4). The hexagonal form appears white because of our inclination to value immediate measurement and the lack of pre-analysis planning.

#### Analytical GREEness metric (AGREE) approach

While there are numerous ways to evaluate an analytical methodology's greenness, only AGREE software employs all 12 GAC principles in its evaluation^44^. Consequently, the method's greenness was assessed using AGREE: The Analytical Greenness Calculator. The clock-like pictogram for the suggested spectrofluorimetric method is shown in Table 4, with an AGREE score of 0.70. The AGREE assessment comparison of the suggested and reported methods reveals that the proposed method is more sustainable than the reported one^26^.

#### Blue Applicability Grade Index (BAGI)

One new metric tool for assessing an analytical method's practicality is the BAGI tool. BAGI is primarily concerned with the applications of White Analytical Chemistry and can be seen as a supplement to the well-established green metrics^45^. An asteroid pictogram as a graphical representation and a corresponding numerical score at the center are the two separate sets of results that the BAGI metric tool offers. The asteroid pictogram, which is made up of different shades of blue to represent varying degrees of compliance (dark blue for high, blue for moderate, light blue for low, and white for non-compliance), serves as a visual representation of the assessment result. Crucially, both methods yield different scores because the pictogram's level of blueness varies. Several sections of the BAGI results for the proposed and reported procedures are subject to a thorough analysis that reveals positive aspects of the "BLUE" criteria^45^. The proposed method displays higher BAGI score along with more dark blue sections than the reported HPLC method^26^, as presented in Table 4.

## RGB12 algorithm for whiteness evaluation

Nowak et al. updated the White Analytical Chemistry method in 2021^37^. The WAC aims to provide an exclusive conceptual tool for incorporating sustainable development principles into analytical chemistry. The idea of sustainable development, which is currently being expanded globally, is multifaceted. i.e., it highlights how crucial it is to try to strike a balance between environmental preservation and research validity, which is linked to the advancement of science. Including green and any other remaining requirements, this metric would enable one to specify all significant expectations formally and methodically for desired technique characteristics. The pillars are composed of three complementary sections. Four specific criteria evaluating fundamental concepts of the analytical method are associated with each section, denoted by a distinct color (red, green, blue). The method's white color is achieved by combining the colors mentioned above. The red section of the study evaluates the analytical effectiveness by employing four algorithms. These algorithms include R1, which assesses the application scope, R2, which examines the limits of detection (LOD) and limits of quantification (LOQ), R3, which evaluates accuracy, and R4, which measures precision. The green section employs four different algorithms to evaluate the green environmental impact. The G1 algorithm assesses the toxicity of reagents, the G2 algorithm calculates the amount of waste and reagents, the G3 algorithm classifies energy and other media, and the G4 algorithm evaluates the direct effects on people, animals, and genetic naturalness. The blue section discusses the influence of four algorithms on practical usefulness and economic conditions. B1 focuses on cost-effectiveness, B2 on time efficiency, B3 on method requirements, and B4 on simplicity of operation. The WAC tool, also known as RGB 12, is associated with the number of rules mentioned earlier. As depicted in Table 4 and Fig S2, both the developed and reported methods are green; however, the developed method has %W more than the reported one.

## Statistical analysis

The proposed spectroflorimetric methods' validity was confirmed by comparing the obtained results for SAF determination in pure form with those obtained using a reported HPLC–UV method^26^. Statistical analysis using the Student t-test and F-test was carried out, the calculated values were found to be lower than the theoretical ones, as shown in Table S3. According to this finding, there is no noticeable difference in the analytical performance between the proposed and reported method.

## Conclusion

As an adjunctive therapy for moderate-to-severe PD, safinamide is newly available in the market. The current study aimed to develop a micelle-mediated spectrofluorimetric method for rapidly quantifying safinamide in the presence of its related precursor impurity, 4-hydroxybenzaldehyde in pharmaceutical dosage forms. The proposed method is a potential alternative to the previously reported ones for routinely analyzing safinamide as it is simple, fast, robust, and environmentally friendly. This method uses green solvents, and incorporating the SDS micellar system which enhanced the method’s sensitivity significantly. The proper choice of a micellar system also allows one to substantially diminish the effects of quenching interferents’ limitation in spectrofluorimetry. Additionally, the high sensitivity of the proposed method allowed for the quantification of SAF in spiked human plasma. Several advanced metric tools were used to assess the method's sustainability, and the results confirmed its excellent greenness and minimal environmental impact. The benefits of the suggested approach have all shown how useful and appropriate it is for use in pharmaceutical quality control labs.

### Supplementary Information

Supplementary Information.

## Data Availability

All data generated or analysed during this study are included in this published article.

## References

[1] Magdy G, Belal F, El-Deen AK (2022). Green synchronous spectrofluorimetric method for the simultaneous determination of agomelatine and venlafaxine in human plasma at part per billion levels. Sci. Rep..

[2] Attia KAM, El-Olemy A, Ramzy S, Abdelazim AH, Hasan MA, Omar MKM, Shahin M (2021). Application of different spectrofluorimetric methods for determination of lesinurad and allopurinol in pharmaceutical preparation and human plasma. Spectrochim. Acta Part A Mol. Biomol. Spectrosc..

[3] Salman, B. I., *et al.* Approach for reduction of the consumed solvent and quantification of avapritinib in biological (2024). 10.1039/d4ra01198h. 10.1039/d4ra01198h PMC10985462 38567336

[4] Wang CC, Silva RA, Masi AN, Fernandez L (2010). Sensitive surfactant-mediated spectrofluorimetric determination of sildenafil. Anal. Methods.

[5] Atia NN, Mahmoud AM, El-Shabouri SR, El-Koussi WM (2013). Two validated spectrofluorometric methods for determination of gemifloxacin mesylate in tablets and human plasma. Int. J. Anal. Chem..

[6] Abdulrazik SG, Attia TZ, Derayea SM (2023). The first spectrofluorimetric protocol for sensitive quantitative analysis of bromocriptine in its pure and pharmaceutical forms: Evaluation of the greenness of the method. RSC Adv..

[7] El-Masry AA, Hammouda MEA, El-Wasseef DR, El-Ashry SM (2020). Eco-friendly green liquid chromatographic separations of a novel combination of azelastine and fluticasone in the presence of their pharmaceutical dosage form additives. Curr. Anal. Chem..

[8] Barakat NT, El-Brashy AM, Fathy ME (2023). Two green spectrofluorimetric methods for the assay of atomoxetine hydrochloride in pure form and commercial capsules with application to content uniformity testing. R. Soc. Open Sci..

[9] Ragab GH, Saleh HM, Abdulla NM, Bahgat EA (2023). Development of green micellar HPLC–DAD method for simultaneous determination of some sulbactam combinations used in COVID-19 regimen. BMC Chem..

[10] Barseem A, Ahmed H, El-Shabrawy Y, Belal F (2019). The use of SDS micelles as additive to increase fluorescence analysis of sitagliptin and saxagliptin derivatives in their tablets and human plasma. Microchem. J..

[11] Derayea SM, Badr El-Din KM, Ahmed AS, Abdelshakour MA, Oraby M (2024). An eco-friendly one-pot spectrofluorimetric approach for the facile determination of overactive bladder drug, tolterodine: Application to dosage forms and biological fluids. Spectrochim. Acta Part A Mol. Biomol. Spectrosc..

[12] Hammad SF, El-Khateeb BZ, El-Malla SF (2021). Micelle-enhanced spectrofluorimetric determination of diphenhydramine: Application to human plasma and its simultaneous determination with naproxen in pharmaceutical tablets. Luminescence.

[13] Mabrouk MM, Noureldin HAM, Badr IHA, Saad AHK (2020). Simple spectrofluorimetric methods for determination of veterinary antibiotic drug (apramycin sulfate) in pharmaceutical preparations and milk samples. Spectrochim. Acta Part A Mol. Biomol. Spectrosc..

[14] Hattori N, Tsuboi Y, Yamamoto A, Sasagawa Y, Nomoto M (2020). Efficacy and safety of safinamide as an add-on therapy to L-DOPA for patients with Parkinson’s disease: A randomized, double-blind, placebo-controlled, phase II/III study. Parkinsonism Relat Disord..

[15] Sanchez Alonso P, De La Casa-Fages B, Alonso-Cánovas A, Martínez-Castrillo JC (2023). Switching from rasagiline to safinamide as an add-on therapy regimen in patients with levodopa: A literature review. Brain Sci..

[16] Huang P, Zhang LY, Tan YY, Di Chen S (2023). Links between COVID-19 and Parkinson’s disease/Alzheimer’s disease: Reciprocal impacts, medical care strategies and underlying mechanisms. Transl. Neurodegener..

[17] Chaná-Cuevas P, Salles-Gándara P, Rojas-Fernandez A, Salinas-Rebolledo C, Milán-Solé A (2020). The potential role of SARS-COV-2 in the pathogenesis of Parkinson’s disease. Front. Neurol..

[18] Cattaneo C, Jost WH, Bonizzoni E (2020). Long-term efficacy of safinamide on symptoms severity and quality of life in fluctuating Parkinson’s disease patients. J. Parkinson’s Dis..

[19] Cruz MP (2017). Xadago (Safinamide) a monoamine oxidase b inhibitor for the adjunct treatment of motor symptoms in Parkinson’s disease. Pharm Ther..

[20] Committee for Medicinal Products for Human Use (CHMP). Assessment report Xadago International non-proprietary name: Safinamide Administrative information (2014).

[21] Zou L, Sun L, Zhang H, Hui W, Zou Q, Zhu Z (2017). Identification, characterization, and quantification of impurities of safinamide mesilate: Process-related impurities and degradation products. J. AOAC Int...

[22] Adhao VS, Thenge RR, Sharma J, Thakare M (2020). Development and validation of stability indicating RP-HPLC method for determination of safinamide Mesylate, Jordan. J. Pharm. Sci..

[23] El-Kosasy AM, Hussein LA, Mohamed NM, Salama NN (2020). New and validated RP-HPLC method for quantification of safinamide mesylate in presence of its basic degradate, levodopa and ondansetron: Application to human plasma. J. Chromatogr. Sci..

[24] Zhang K, Xue N, Shi X, Liu W, Meng J, Du Y (2011). A validated chiral liquid chromatographic method for the enantiomeric separation of safinamide mesilate, a new anti-Parkinson drug. J. Pharm. Biomed. Anal..

[25] Redasani VK, Mali BJ, Patil AS, Shirkhedkar AA (2013). Development and validation of RP-HPLC method for determination of safinamide mesylate in bulk and in tablet dosage form. Anal. Chem. Indian J..

[26] El-Sayed HM, Abdellatef HE, Hendawy HAM, El-Abassy OM, Ibrahim H (2023). DoE-enhanced development and validation of eco-friendly RP-HPLC method for analysis of safinamide and its precursor impurity: QbD approach. Microchem. J..

[27] Redasani VK, Mali BJ, Surana SJ (2012). Development and validation of HPTLC method for estimation of safinamide mesylate in bulk and in tablet dosage form. Int. Sch. Res. Not..

[28] Tammisetty MR, Challa BR, Puttagunta SB (2020). Application of liquid chromatography wtih tandem mass spectrometric method for quantification of safinamide in invitro samples. Int. J. Pharma Bio Sci..

[29] El-sayed HM, Abdel-Raoof AM, Abdellatef HE, Hendawy HAM, El-Abassy OM, Ibrahim H (2022). Versatile eco-friendly electrochemical sensor based on chromium-doped zinc oxide nanoparticles for determination of safinamide aided by green assessment criteria. Microchem. J..

[30] El-Sayed HM, Abdellatef HE, Mahmoud AM, Hendawy HAM, El-Abassy OM, Ibrahim H (2023). Safinamide detection based on Prussian blue analogue modified solid-contact potentiometric sensor. Microchem. J..

[31] El-Sayed HM, El-Abassy OM, Abdellatef HE, Hendawy HAM, Ibrahim H (2023). Green spectrophotometric platforms for resolving overlapped spectral signals of recently approved antiparkinsonian drug (safinamide) in the presence of its synthetic precursor (4-hydroxybenzaldehyde): Applying ecological appraisal and comparative statisti. J. AOAC Int..

[32] Abdel Moneim MM, Hamdy MMA (2021). Green spectrofluorimetric methods for tramadol assay with ibuprofen or chlorzoxazone: Comparison of greenness profiles. Luminescence.

[33] Gałuszka A, Migaszewski Z, Namieśnik J (2013). The 12 principles of green analytical chemistry and the SIGNIFICANCE mnemonic of green analytical practices. TrAC Trends Anal. Chem..

[34] Kurowska-susdorf A, Zwier M, Ivankovi A (2019). Green analytical chemistry: Social dimension and teaching. Trends Anal. Chem..

[35] Lotfy HM, El-Hanboushy S, Fayez YM, Abdelkawy M, Marzouk HM (2024). Computational intelligence spectrophotometric scenarios for screening and quantification of single-dose triple therapy banned by the World Anti-Doping Agency in some sports. Microchem. J..

[36] Wadie M, Abdel-moety EM, Rezk MR, Marzouk HM (2023). A novel eco-friendly HPLC method with dual detection modes for versatile quantification of dutasteride and silodosin in pharmaceutical formulation, dissolution testing and spiked human plasma. Microchem. J..

[37] Nowak PM, Wietecha-Posłuszny R, Pawliszyn J (2021). White analytical chemistry: An approach to reconcile the principles of green analytical chemistry and functionality. TrAC Trends Anal. Chem..

[38] Rostom Y, Rezk MR, Wadie M, Abdel-Moety EM, Marzouk HM (2024). State-of-the-art mathematically induced filtration approaches for smart spectrophotometric assessment of silodosin and solifenacin mixture in their new challenging formulation: Multi-tool greenness and whiteness evaluation. Spectrochim. Acta Part A Mol. Biomol. Spectrosc..

[39] Sajid M, Justyna P (2022). Green analytical chemistry metrics: A review. Talanta.

[40] Hammad SF, El-Malla SF, El-Khateeb BZ (2023). Enhanced fluorimetric detection of diphenylpyraline HCl using micelle and cyclodextrin mediated approach: Spectrofluorimetric and micellar liquid chromatographic application for either single or combined formulation with caffeine and paracetamol. Spectrochim. Acta Part A Mol. Biomol. Spectrosc..

[41] ICH Harmonized Tripartite Guidline. Validation of Analytical Procedures: Text and Methodology Q2 (R1) (2005)

[42] Kokosa JM, Przyjazny A (2022). Green microextraction methodologies for sample preparations. Green Anal. Chem..

[43] Płotka-Wasylka J, Wojnowski W (2021). Complementary green analytical procedure index (ComplexGAPI) and software. Green Chem..

[44] Pena-Pereira F, Wojnowski W, Tobiszewski M (2020). AGREE—analytical GREEnness metric approach and software. Anal. Chem..

[45] Manousi N, Wojnowski W, Płotka-Wasylka J, Samanidou V (2023). Blue applicability grade index (BAGI) and software: A new tool for the evaluation of method practicality. Green Chem..

