# Supplementary material for: Ecofriendly micellar mediated spectrofluorimetric method for ultrasensitive quantification of the antiparkinsonian drug safinamide in pharmaceutical formulation and spiked human plasma
Source: Sci Rep. 2024 Jul 16;14:16460. doi: 10.1038/s41598-024-66462-7 (PMC11252346; doi:10.1038/s41598-024-66462-7)
Supplement: Supplementary file 1 — Supplementary Information. [file 41598_2024_66462_MOESM1_ESM.docx]

**Fig S1.** The linearity plot of SAF over the concentration range of 10.0-1000.0 ng/mL against the relative fluorescence intensities.


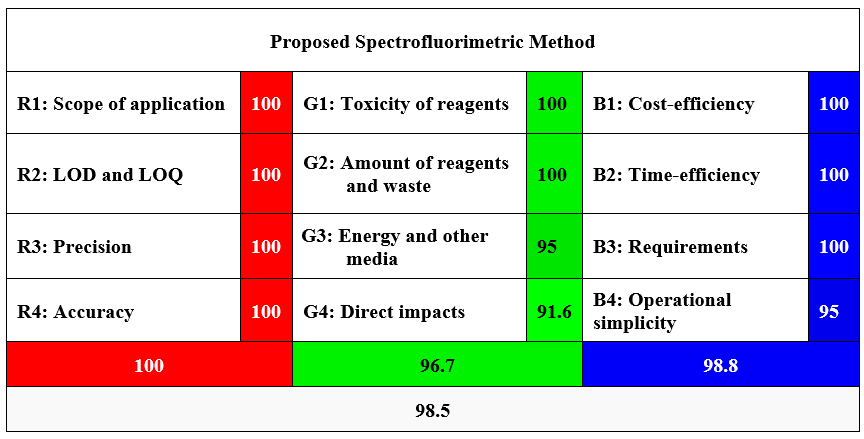


**(a)**

**(b)**

**Fig S2.** Whiteness assessment of a) the proposed spectrofluorimetric method and b) the reported HPLC-UV method [26].

**Table S1.** The comparison for LODs of the proposed method with the previously reported ones.

| Method | LOD (ng/mL) | Reference |
| --- | --- | --- |
| The proposed spectrofluorimetric method | 1.91 |  |
| Reported HPLC–UV method | 27.00 | [21] |
| Reported HPLC-PDA method | 150.00 | [22] |
| Reported HPLC-UV method | 110.00 | [23] |
| Reported HPLC-UV method | 15.00 | [24] |
| Reported HPLC-UV method | 270.00 | [25] |
| Reported HPLC-UV method | 950.00 | [26] |
| Reported HPTLC-Densitometric method | 13.09 | [27] |
| Reported UPLC-MS/MS method | 0.012 | [28] |
| Reported Voltammetric method | 162.00 | [29] |
| Reported Potentiometric method | 3586.50 | [30] |
| Reported spectrophotometric method | 598.00 | [31] |

**Table S2.** Selectivity of the proposed native spectrofluorimetric method for determining safinamide in laboratory-prepared mixtures with its related impurity.

| 4-HBD  %(w/w) | Recovery ^a^ % |
| --- | --- |
|  | **SAF** |
| 1% | 98.42 |
| 2% | 100.17 |
| 4% | 98.30 |
| 5% | 101.47 |
| 6% | 98.50 |
| Mean ± SD | **99.37 ± 1.402** |

^a^ Average of three determinations.

**Table S3.** Statistical comparison of the results obtained by the proposed spectrofluorimetric method and the reported method for determination of safinamide in pure form.

| Parameters | Spectrofluorimetric method | Reported method ^a)^ |
| --- | --- | --- |
|  | **SAF** | **SAF** |
| Mean | 100.01 | 100.05 |
| SD | 1.453 | 1.436 |
| n | 5 | 5 |
| Variance | 2.111 | 2.063 |
| Student’s t-test (2.306) ^b)^ | 0.48 | ------- |
| F value (6.39) ^b)^ | 1.02 | ------- |

1. HPLC method using Waters VDSpher PUR 100 C_18_-E column (5 µm, 250 ×4.6 mm) as analytical column, mobile phase consists of a mixture of f 0.02 M ammonium formate and 0.02 M ammonium acetate, with the pH adjusted to 5.5 using formic acid and ethanol (60:40, v/v) at a flow rate of 1 mL/min and detected at 220.0 nm [26].
2. Tabulated t- and F values at P = 0.05.
